# Sequence analysis of LipL41 and LipL21: Prospective Outer Membrane Proteins (OMPs) in early diagnosing leptospirosis

**DOI:** 10.1016/j.mex.2022.101804

**Published:** 2022-07-30

**Authors:** Muzaffar Mosquill, Syafinaz Amin Nordin, Mohamad Ridhuan Mohd Ali, Narcisse Mary Sither Joseph

**Affiliations:** a Department of Medical Microbiology, Faculty of Medicine and Health Sciences, Universiti Putra Malaysia, Selangor 43400, Malaysia; b Ministry of Health, Institute for Medical Research, Malaysia

**Keywords:** Leptospirosis, *Leptospira*, Bioinformatics, PCR, Lipoproteins, LipL21, LipL41

## Abstract

Leptospirosis is a zoonotic disease mostly occurring in tropical climate countries. The etiology of the disease is due to microbes from the genus *Leptospira*. Higher number of cases reported worldwide indicated the disease is not easily eradicated. Leptospirosis shares the most common febrile symptoms such as dengue, Zika and yellow fever thus making it difficult to differentiate the disease at an early stage. The widely used current detection via PCR, uses the bacterial outer membrane protein (OMP) as their target region. However, the heterogeneity and variation of the genome cause false negative results. Lipoprotein LipL41 is the third most abundant outer membrane lipoprotein among pathogenic species and it is surface exposed and expressed during infection thus making it a suitable candidate in identifying pathogenic *Leptospira*. LipL21 on the other hand is a potential candidate in identifying the intermediate species. The study aimed in designing suitable PCR primers in identifying pathogenic and intermediate species of *Leptospira* through bioinformatics analysis on the bacterial OMPs. LipL41 and LipL21 were chosen as the suitable target sequence to be used as PCR primers in detecting the pathogenic and intermediate species, respectively. The designed primers indicated positive feedback upon tested with their respective bacterial DNA extract. These lipoproteins may serve as potential PCR primers to be used with clinical samples in diagnosing leptospirosis.

• The etiology of the illness is due to bacteria from the genus *Leptospira*.
• PCR utilizes the bacterial external membrane protein (OMP) thus the heterogeneity and variety of the genome cause bogus adverse outcomes.
• The suitable candidates are LipL41, the third most abundant outer membrane lipoprotein, whereas LipL21 is a potential candidate in identifying the intermediate species.

## Graphical abstract

**Figure:**
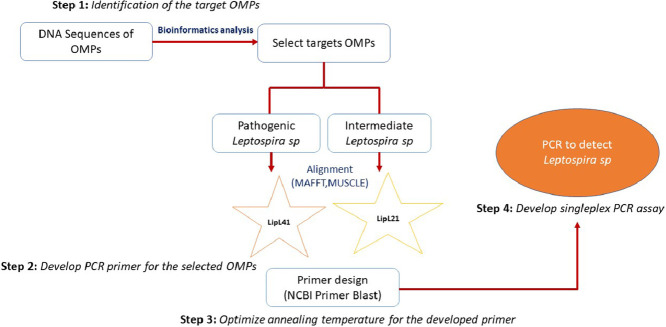

Specifications table

**Table:** 

Subject area:	Biochemistry, Genetics and Molecular Biology
More specific subject area:	*Polymerase Chain Reaction, PCR*
Name of your method:	*Sequence Analysis on LipL21 and LipL41*
Name and reference of original method:	Yap, M. L., Sekawi, Z., Chee, H. Y., Ong, H. A., & Neela, V. (2019). Comparative analysis of current diagnostic PCR assays in detecting pathogenic Leptospira isolates from environmental samples. *Asian Pacific Journal of Tropical Medicine, 12*(10), 472–478. https://doi.org/10.4103/1995-7645.269908
Resource availability:	• NCBI Genome Database (https://www.ncbi.nlm.nih.gov/genome/) • NCBI Primer Blast (https://www.ncbi.nlm.nih.gov/tools/primer-blast)

## Method details

**Fig. 1:**
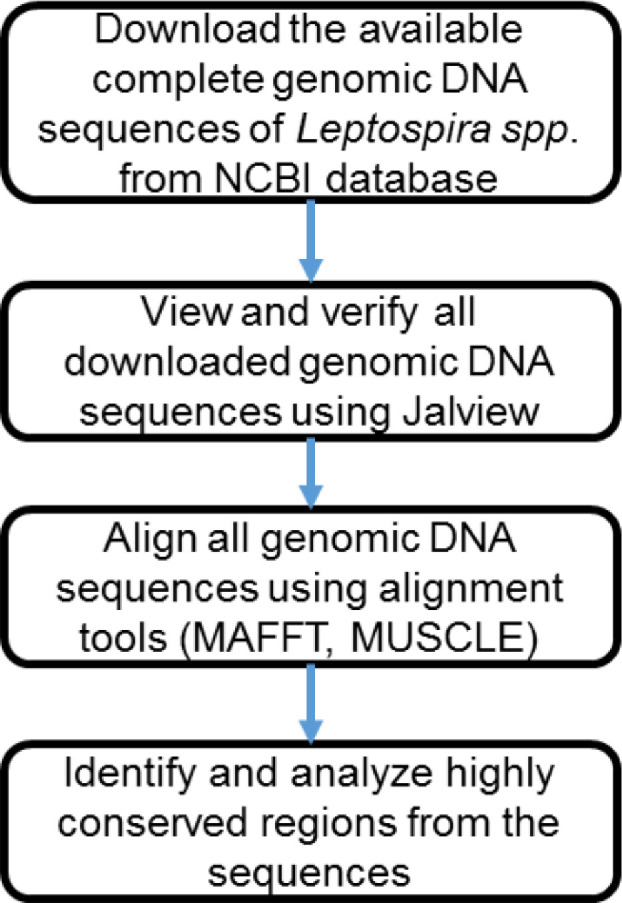
Process flow in identification of the OMP conserved sequences.

The study methodology is categorized into 4 steps which includes identification of the conserved sequences in leptospiral OMPs. This was achieved by downloading the selected (LipL21, LipL31, LipL41, LipL46) leptospiral OMP sequences that are available in the National Center for Biotechnology Information, NCBI database. The sequences were later aligned with multiple sequence alignment tool Multiple Sequence Comparison by Log-Expectation (MUSCLE) and viewed using bioinformatics tools such as Jalview. Once aligned, the sequences were analyzed for their percentage for identity and the conserved sequences available. The flow of the process is simplified as shown in Fig. 1. The higher conserved sequences available across the respective bacterial species indicated higher similarities among them. Bioinformatics analysis on the sequences of Leptospiral OMPs revealed the presence of conserved regions in LipL21 among the intermediate species of *Leptospira* and LipL41 among pathogenic species. Therefore, we chose LipL21 and LipL41 as the target gene for detecting the intermediate and pathogenic species, respectively.

The second step is to develop PCR primer from the conserved sequence based on the highly conserved sequence observed previously. Lipoprotein LipL41 and LipL21 were suitable for the target sequence in detecting the pathogenic and intermediate species of *Leptospira*, respectively. The target sequence will be developed into PCR primers. The primers are developed using primer designing tools such as NCBI Primer Blast (https://www.ncbi.nlm.nih.gov/tools/primer-blast). Sequences that will be used for the primer assembly are listed in Table 1.

**Table 1:** List of target sequences that were used in designing the PCR primer.

No	Target	Species	Forward Sequence (5’ → 3’)	Reverse Sequence (5’ → 3’)	Amplicon Size (base pair, bp)
1	LipL21	*L. hartskeerli* strain MCA1-C-A1	ATG GTC TTT CGA AGG CTG GG	GTT GCT TCA CCG TCG GAA AC	258 bp
2	LipL41	*L. interrogans* strain FDAARGOS	CGT AAC GTA GGT TTG GCT GT	TGC TTC GTT GAT TGC GTC TG	174 bp

Several cultures of *Leptospira* species are selected to be used in this study to validate the effectiveness of the developed primer in detecting the correct *Leptospira* species. The selection comprises of several species according to the pathogenicity of the bacteria. The *Leptospira* species are cultured in EMJH media and the cultures are regularly observed under the dark field microscopy to observe their progress. The cultures are then proceeded with DNA extraction using Nucleospin Tissue DNA extraction kit (Macherey Nagel, Germany). The concentration of the extracted bacterial DNA was quantified using Nanodrop Spectrophotometer (Thermo Fisher Scientific, USA) and were diluted to 10µg/mL with deionized RNAse free water for PCR.

The third step is to optimize the annealing temperature of the developed primer. This can be done by performing temperature gradient on the developed primer. This step is crucial as we need to identify the suitable annealing temperature of each primer. In this study, we select ranges of between 50 and 60 ⁰C for the temperature gradient. Each primer is optimized through conventional PCR with temperature gradient to identify the suitable annealing temperature using exTEN 2X PCR Master Mix (1st Base, Singapore). As many as eight (8) reaction mix are prepared according to Table 2 for each primer set with the DNA template extracted from bacteria cultures of *L. fainei* serovar Hurstbridge and *L. interrogans* serovar Icterohaemorrhagiae to be used on LipL21 and LipL41 primer, respectively. The mastermix reaction mixture were later dispensed equally into 8 PCR vessels or tubes labeled A, B, C, D, E, F, G and H making the total volume in each PCR reaction vessel or tube approximately 25 μL. The PCR tubes are covered and placed inside the thermal cycler (Bio-Rad CFX96, USA) with the cycle programmed according to the conditions stated in Table 3. Once the minimum and maximum annealing temperature were confirmed based on the melting temperature of the primers (should be less than the Tm of primers), the temperature in each of the PCR tubes will be set by the thermal cycler as indicated in Table 4. The suitable annealing temperature are selected according to the density or presence of amplicon in the gel electrophoresis for both primers during the temperature gradient.

**Table 2:** PCR reaction mix solution for primer's temperature gradient.

No.	Component	Volume / reaction (µL)	Volume for 8X reaction (µL)
1	exTEN 2X PCR Master Mix	12.5	100
2	Primer (Forward) (10 µM)	1.5	12
3	Primer (Reverse) (10 µM)	1.5	12
4	RNase-Free Water / sterilized distilled H_2_O	7.5	60
5	DNA Template	2	16
**Total Reaction Volume**		**25**	**200**

**Table 3:** Thermal cycler conditions for temperature gradient.

No	Step	Temperature (⁰C)	Time (seconds)
1	PCR initial activation step	95	60
2	Denaturation	95	15
3	Annealing	50-60	30
4	Extension	72	60
5	Final Extension	72	600
6	Number of cycles	35 cycles

**Table 4:** The temperature gradient setting for each of the PCR tubes.

PCR vessel/tube	A	B	C	D	E	F	G	H
Temperature (⁰C)	60.0	59.4	58.3	56.3	53.9	52.0	50.7	50.0

**Fig. 2:**
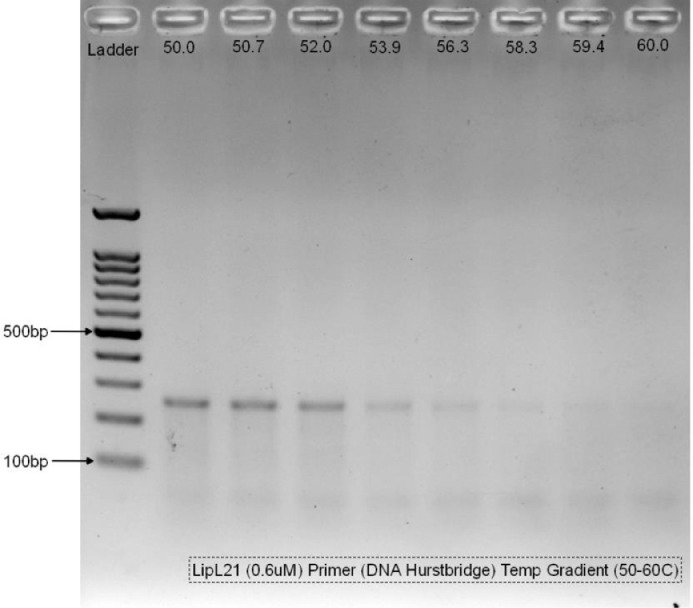
Temperature gradient on LipL21 primer.

**Fig. 3:**
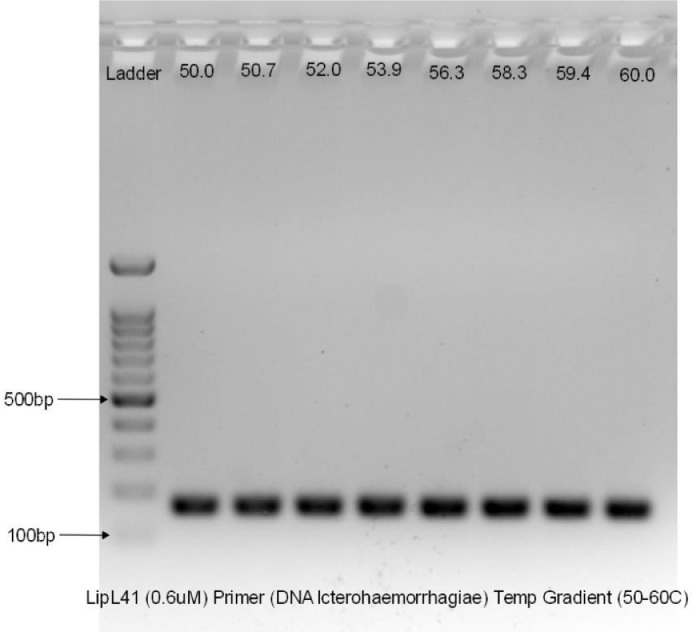
Temperature gradient on LipL41 primer.

To visualize the PCR product, we need to perform gel electrophoresis. Agarose gel with concentration of 1.8% were prepared by mixing 1.8 g of agarose powder with 100 mL of 1X Tris-Acetate-EDTA (TAE) buffer (Thermo Scientific, Lithuania). The mixed solution is heated up in a microwave oven for approximately 2 to 3 min to dissolve the agarose powder and left at room temperature to cool down. Once cooled down, 1 µL of EtB”Out” nucleic acid staining solution (Yeastern Biotech, Taiwan) were added into the agarose solution. The nucleic acid staining solution is a substitute to Ethidium Bromide which is essential for the nucleic acid detection in agarose gels. The solution is gently poured into a gel tray with combs to form the wells. The hardened gel is then placed and submerged into the electrophoresis tank containing 1X TAE buffer solution. The gel is aligned with the well facing the negative terminal of the electrophoresis tank. About 5 µL of Excelband 100bp DNA ladder (Smobio, Taiwan) were loaded into the well using a micropipette and followed by the same volume of the PCR products from the second well onwards. The gel electrophoresis was run at 100 V for 45 min. Once complete, the gel was placed onto the imaging system (Bio-Rad GelDoc Go Gel, USA) to visualize the result as shown in Fig. 2, Fig. 3.

The final step of the study is to develop singleplex PCR assay for each primer designed. The assay shall be tested with their respective negative and positive control as well as the DNA taken from saprophytic (*L. biflexa* serovar Patoc), intermediate (*L. licerasiae* serovar Varillal) for LipL21; (*L. fainei* serovar Hurstbridge) for LipL41 and pathogenic (*L. weilii* serovar Caledonia) species of *Leptospira*. The negative control used are the mastermix reaction mixture with the DNA template substituted with sterilized distilled H_2_O. The positive control used are the mastermix reaction mixture with DNA template *L. fainei* serovar Hurstbridge for LipL21 and *L. interrogans* serovar Icterohaemorrhagiae for LipL41 which previously used during temperature gradient reaction mixture. In this study, we select the suitable annealing temperature of 52.0 ⁰C. The PCR products of both assays were run on 1.8% agarose gel electrophoresis at 100 V for 45 min.

**Fig. 4:**
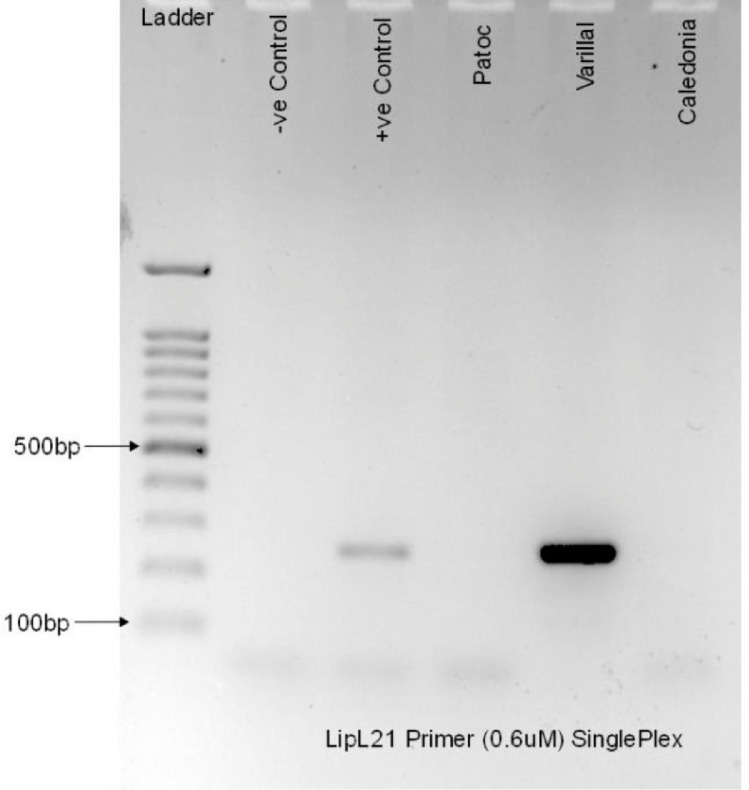
Singleplex run on the developed LipL21 primer.

**Fig. 5:**
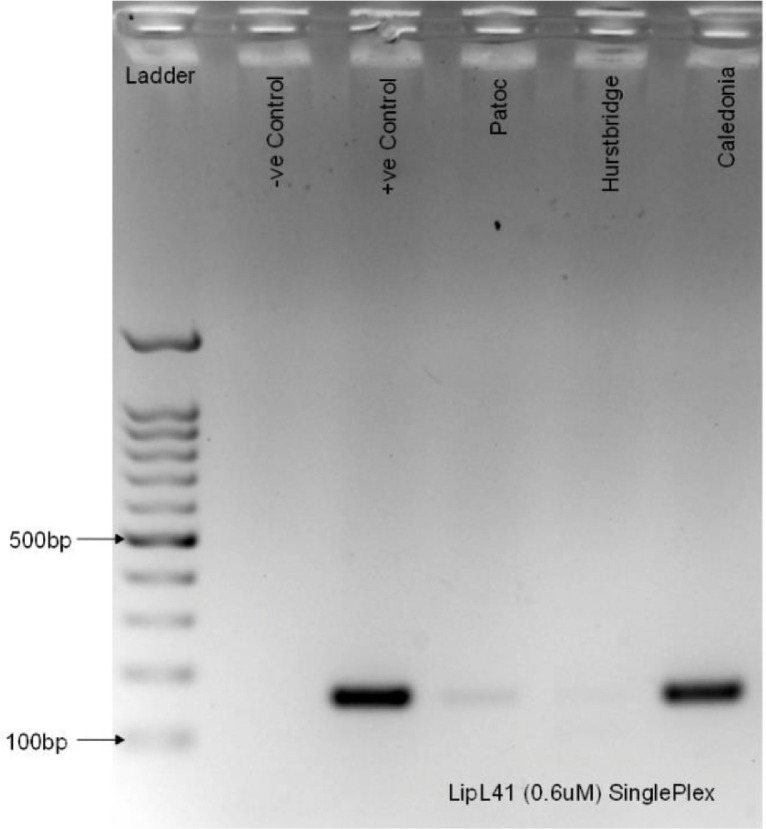
Singleplex run on the developed LipL41 primer.

The outcome of the study, we identified that our developed LipL21 primer producing amplicon located between 200 and 300 base pair (bp) within the intermediate species (DNA Varillal) and not seen within the pathogenic species (DNA Caledonia) and saprophytic species (Patoc) as shown in Fig. 4. We also identified that the developed LipL41 primer indicated presence of amplicon located between 100 and 200bp on DNA template Patoc and Caledonia as shown in Fig. 5. The size of the amplicon is approximately 174bp. However, there is no amplicon indicated on DNA template Hurstbridge in LipL41.

Leptospirosis should not be taken easily as reports indicated that the incidence rate pertaining to this disease is increasing annually [6,7]. Most common method used in diagnosing the disease is MAT [5]. Nevertheless, certain region or states incorporates PCR to overcome the shortcomings of MAT. However, a study conducted by Joseph et al. [4] indicated that sensitivity of LipL32 which is commonly used in detecting pathogenic species is only 33.9% compared to MAT. Nowadays, there are more than 250 serovars have been identified worldwide [8]. Most of studies pertaining in diagnosing the disease are focusing in identifying the pathogenic species of *Leptospira* whereby several studies indicated the intermediate species could cause the higher number of cases [1,2]. Therefore, the PCR primers that are being used today in identifying the pathogen requires optimization to address the issues.

Based on this study, we found out that LipL21 and LipL41 shows eminent relationship between the pathogenic and intermediate species thus they are suitable OMP candidates to be used in differentiating between the pathogenic and intermediate species of *Leptospira*. Through bioinformatics analysis of both sequences of LipL21 and LipL41, we are able to develop PCR primers as mentioned by Cosate et al. [3] that can be used in detecting the pathogenic and intermediate species. Although the developed primers were not clinically tested, but these findings will enhance future studies that will soon find its way to be used in clinically diagnosing leptospirosis.

## Ethics statements

MethodsX has ethical guidelines that all authors must comply with. In addition, we ask you to complete the relevant statement(s) below. Please delete those which are not relevant to your work.

The study is neither involving human subjects, animal experiments nor data collected from social media platforms.

## CRediT authorship contribution statement

**Muzaffar Mosquill:** Conceptualization, Methodology, Investigation, Writing – original draft. **Syafinaz Amin Nordin:** Supervision. **Mohamad Ridhuan Mohd Ali:** Validation, Supervision. **Narcisse Mary Sither Joseph:** Visualization, Data curation, Supervision, Writing – review & editing.

## Declaration of Competing Interest

None.

## Data Availability

No data was used for the research described in the article.
